# Withdrawal of inhaled corticosteroids in individuals with COPD - a systematic review and comment on trial methodology

**DOI:** 10.1186/1465-9921-12-107

**Published:** 2011-08-12

**Authors:** Nighat J Nadeem, Stephanie JC Taylor, Sandra M Eldridge

**Affiliations:** 1 Centre for Health Sciences, Barts and The London School of Medicine and Dentistry, Queen Mary University of London 2 Newark Street, London, E1 2AT, UK

## Abstract

Inhaled corticosteroids (ICS) reduce COPD exacerbation frequency and slow decline in health related quality of life but have little effect on lung function, do not reduce mortality, and increase the risk of pneumonia. We systematically reviewed trials in which ICS have been withdrawn from patients with COPD, with the aim of determining the effect of withdrawal, understanding the differing results between trials, and making recommendations for improving methodology in future trials where medication is withdrawn. Trials were identified by two independent reviewers using MEDLINE, EMBASE and CINAHL, citations of identified studies were checked, and experts contacted to identify further studies. Data extraction was completed independently by two reviewers. The methodological quality of each trial was determined by assessing possible sources of systematic bias as recommended by the Cochrane collaboration. We included four trials; the quality of three was adequate. In all trials, outcomes were generally worse for patients who had had ICS withdrawn, but differences between outcomes for these patients and patients who continued with medication were mostly small and not statistically significant. Due to data paucity we performed only one meta-analysis; this indicated that patients who had had medication withdrawn were 1.11 (95% CI 0.84 to 1.46) times more likely to have an exacerbation in the following year, but the definition of exacerbations was not consistent between the three trials, and the impact of withdrawal was smaller in recent trials which were also trials conducted under conditions that reflected routine practice. There is no evidence from this review that withdrawing ICS in routine practice results in important deterioration in patient outcomes. Furthermore, the extent of increase in exacerbations depends on the way exacerbations are defined and managed and may depend on the use of other medication. In trials where medication is withdrawn, investigators should report other medication use, definitions of exacerbations and management of patients clearly. Intention to treat analyses should be used and interpreted appropriately.

## Review

### Introduction

The mortality, morbidity and economic burden of Chronic Obstructive Pulmonary Disease (COPD) exacerbations is well documented [1-4]. Reduction in the frequency of exacerbations is a major therapeutic aim in COPD and treatment with inhaled corticosteroids (ICS) has been associated with a 25% reduction in exacerbations [5]. In a Cochrane review by Yang, ICS were found both to reduce the frequency of COPD exacerbations by 0.26 per patient per year (weighted mean difference: 95% CI -0.37 to -0.14) and to slow the rate of decline in health related quality of life as determined by the St George's Respiratory Questionnaire (weighted mean difference: -1.22 units/year, 95% CI -1.83 to -0.60) [6]. Despite this, Yang's review failed to demonstrate any effect of ICS on the decline in lung function or on mortality, although a more recent paper identifies a small clinically insignificant effect on lung function [7]. It is now recognised that ICS in stable COPD are associated with an increased risk of pneumonia (relative risk 1.34 95% CI, 1.03-1.75) [8], and the long-term use of high-dose ICS has been associated with adverse effects including cataracts [9], glaucoma [10] and osteoporosis [11]. UK National Institute of Health and Clinical Excellence (NICE) guidance discourages the use of ICS as monotherapy, but does encourage their use with bronchodilators if patients have moderate or severe COPD and are still symptomatic, or are experiencing two or more exacerbations requiring treatment per year [3].

In light of the debate around the role of long term ICS in COPD [8], consideration of the potential effects of withdrawing ICS becomes important, but there are no reviews of withdrawing treatment. The aims of this systematic review were to determine the effects of withdrawal of ICS, to use the review process to explore the reasons for different results between trials, and to make recommendations for improving methodology in future trials where medication is withdrawn.

## Method

### Search strategy and selection criteria

The search strategy involved text words for COPD and the names of inhaled steroids used in COPD and relevant Medical Subject Headings (MeSH). Boolean logic was used to truncate and combine terms and the search strategy was modified several times before being finally performed (full search available from the authors). The Cochrane Library and the Database of Abstracts and Reviews were searched for existing systematic reviews on this topic. None were found so we searched for published original articles in PubMed, EMBASE and CINAHL from inception to February 2007. Criteria for the inclusion and exclusion of studies were based on the four facets: Population, Intervention, Comparison and Outcome. Studies were included if they were randomised controlled trials, comparing patients withdrawn from ICS with those not withdrawn, in which the diagnostic criteria for COPD were consistent with GOLD, NICE or ATS [1,3,12]. Studies were included if they assessed any of the following outcome measures: lung function, exacerbations, health related quality of life, exercise tolerance and the use of healthcare facilities because of respiratory symptoms. Due to time and resource constraints it was not possible to include non-English language papers.

### Procedures

Two reviewers independently reviewed papers identified by the electronic search for potential inclusion on the basis of their titles and abstracts and independently scrutinised full texts of papers considered potentially includable to confirm inclusion/exclusion. Any discrepancies were resolved through discussion between the two reviewers. To identify further studies, we searched citations of included reports and contacted experts in the field. Data from each included trial were extracted independently by two reviewers. Again, disagreements were resolved through discussion. We extracted data on outcomes, the design and conduct of the trial, the way in which exacerbations were defined, the protocol for managing severe exacerbations, and the methodological quality of the trials.

### Trial quality

To assess quality we used the Cochrane collaboration classification scheme for assessing bias [13]. We used the following criteria: method of randomisation, sequence generation, and allocation concealment for selection bias; blinding for performance bias; whether the loss of patients were accounted for and whether the analysis was by intention to treat for attrition bias; whether the outcome assessor was blinded for detection bias, and whether there was evidence of selective reporting of outcome measures for reporting bias.

### Analysis

We only conducted a meta-analysis on outcome data from trials which we rated as acceptable in terms of bias on at least three of the five bias criteria listed above, and only when relevant data could be obtained from at least three trials. For other outcomes we report results from individual trials. We meta-analysed outcome data using the random effects method of DerSimonian & Laird [14], with the estimate of heterogeneity being taken from the Mantel-Haenszel model and assessed for statistical significance using a Cochran Q-test which follows a chi-squared distribution. All statistical analyses were performed in Stata, version 10.1. We identified features of the included trials that we felt were important in interpreting results. Based on an examination of the treatment of these features in the included papers, we make recommendations for the future design of these types of trial. Statistical significance was reported at the 5% level in all trials and we report all results at that level.

## Results

**Figure 1:**
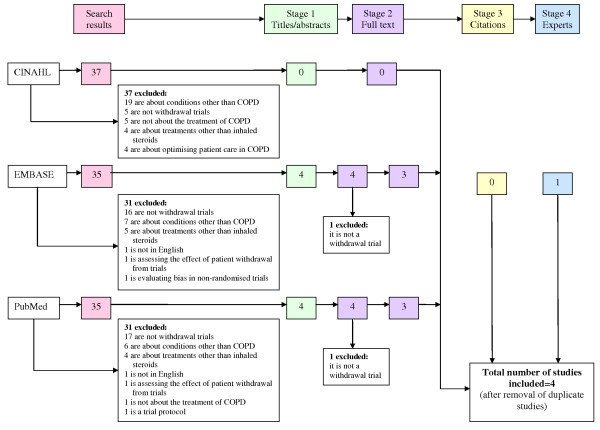
**Summary of the research process**.

The electronic search identified 107 publications of which 103 were excluded on the basis of their titles and abstracts (Figure 1). The full texts of four papers were retrieved. One was excluded because it did not compare patients withdrawn from ICS with those not withdrawn. A further trial published during the course of this research was included; one of the authors of the present paper is an author on this trial. Searching through references of these trials or contacting experts did not identify any further studies.

Characteristics of the included trials are shown in Table 1 and characteristics of the patients included in Table 2. The number of patients in these trials ranged from 24-615. Three of the trials were parallel group trials (COPE, COSMIC, WISP) [15-17] ranging in duration from 10-12 months with a run-in period of between 2 weeks and 4 months. The reason for a run-in is usually to screen patients so that those who are unsuitable for the trial can be identified and excluded before randomisation. In two of the trials, however, the run-in period involved all patients being administered the same inhaled corticosteroid treatment, and in the COSMIC trial authors explain the length of their run-in, which involved all patients being put on the same treatment as 'to be on the safe side of the GOLD guidelines which recommend a trial period of 6 weeks to 3 months to get a plateau in the response to FEV_1 _to ICS'. The fourth trial was a cross-over trial with a total duration of 12 weeks and no run-in period [18]. The parallel trials used fluticasone as the inhaled steroid but the cross-over trial used beclomethasone. During the run-in period, WISP patients received their usual medication, COSMIC and COPE patients were given the inhaled steroid which was to be used for the remainder of the trial.

**Table 1:** Description of studies

*Trial/Country/Publication date*	*Type of study*	*Duration & follow-up*	*Number of patients randomised*	*Run-in period*		*Trial period medication*		*Outcomes*
					
				Duration	Treatment	Steroid group	Withdrawn group	
*WISP^14^/*United Kingdom/2007	Parallel	12 months (follow up every 3 months)	260	2 weeks	Patient's usual medication	FP 500 ug twice daily	Placebo	Exacerbation frequency* Time to first exacerbations Respiratory symptoms PEFR Reliever inhaler use Lung function HRQL
*O'Brien^17^/USA/2001*	Crossover	12 weeks (follow up every 3 weeks)	24	None	n/a	BDP 84 ug 4 times daily	Placebo	Lung function* Exercise capacity* HRQL Sputum analysis
*COPE^15^/Netherlands/2002*	Parallel	6 months (follow up at 3& 6 months)	244	4 months	FP 500 ug twice daily & ipratropium bromide 40 ug four times daily	FP 500 ug twice daily and Ipratropium	Placebo and Ipratropium	First and second exacerbations* Rapid recurrent exacerbation* HRQL* Lung function Exercise tolerance Use of healthcare facilities Respiratory symptoms
*COSMIC^16^/Netherlands/2005*	Parallel	12 months (follow up at weeks 0, 4, 11, 12, 16, 28, 40, 52, 64 & 66)	373	3 months	Combined salmeterol 50 ug & fluticasone 500 ug twice daily	Combined salmeterol 50 ug & fluticasone 500 ug twice daily	Salmeterol 50 ug twice daily	Lung function* Exacerbation & use of rescue medication Respiratory symptoms HRQL

*Primary outcome measures.

**Table 2:** Patient characteristics

*Study*	*Age *	*Sex*	*Baseline severity of COPD *	*Duration of inhaled steroid use prior to entry into trial*	*Mean baseline FEV^1^*	*Mean baseline number of exacerbations in year preceding trial*
*WISP^14^*	> 40	Male & female	Post-bronchodilator FEV1 < 80% predicted	Minimum 6 months, mean 8 years	Withdrawn group: 1.40 L Steroid group: 1.31 L (post-bronchodilator)	Withdrawn group: 1.86 Steroid group: 1.93
*O'Brien et al^17^*	40-79	Male	Details not provided	Details not provided	1.61 L	Details not provided
*COPE^15^*	40-75	Male & female	Pre-bronchodilator FEV1 25-80% predicted	83% of patients had used for at least 6 months	Withdrawn group: 1.69 L Steroid group: 1.78 L (post-bronchodilator)	All patients: 1.3
*COSMIC^16^*	40-75	Male & female	Pre-bronchodilator FEV1 30-70% predicted	Details not provided	Withdrawn group: 49.0 Steroid group: 48.1 (pre-bronchodilator % predicted)	Details not provided, all patients had 2 or more requiring treatment

The three parallel trials all had an acceptable level of bias for three or more bias criteria (Table 3). The fourth trial appeared to have a much greater potential for bias, was substantially different in size and design, and had a different active treatment. We did not consider data from this trial for our meta-analyses. Thus we could only meta-analyse data from a maximum of three trials. For most of the outcomes we considered, data were either not reported or not reported clearly for at least one trial. As a result we only conducted a meta-analysis for one outcome: whether or not a patient had an exacerbation during the study period.

**Table 3:** The methodological quality of the trials

*Type of bias*	*Criteria*	*WISP*			*O'Brien et al*	*COPE*			*COSMIC*		
**Selection**	Method used to generate randomisation sequence?	'Patients were allocated with minimisation to intervention using the programme MINIM v1.3'			'Randomisation was performed by the clinical pharmacist who randomised an odd number dice roll to placebo and an even number dice roll to drug'	'Randomisation was performed in blocks of six by computer generated allocation'			'A randomisation schedule generated by the patient allocation for clinical trials (PACT) program'		
	Method used to generate allocation concealment?	'Inhalers were given an alphanumeric code to conceal allocation'			Inadequate	Unclear			Unclear		
**Performance**	Double-blinding?	'Study nurses and regular clinicians were blind to allocation throughout the study'			'The subject and pulmonary physician were blinded to the treatment regimen. Placebo and drug MDI canisters were identical, and the placebo mist was flavoured to make the treatments indistinguishable'	'This study was a randomised, double-blind parallel-group single centre study...'			'the study medication was packed in identical inhaler devices to ensure both the patient and investigator were unaware of the allocated treatment'		
**Attrition**	Loss of patients accounted for?		Steroid	Placebo	Unclear		Steroid	Placebo		Steroid	Placebo
		Number randomised	128	132		Number randomised	123	121	Number randomised	189	184
		Number analysed	128	132		Number analysed	122(1 died)	120(1 died)	Number analysed	unclear	unclear
**Detection**	Outcome assessor blind	Unclear			Unclear	Unclear			Unclear		
**Reporting**	Any evidence of reporting bias?	No			No	No			No		

**Figure 2:**
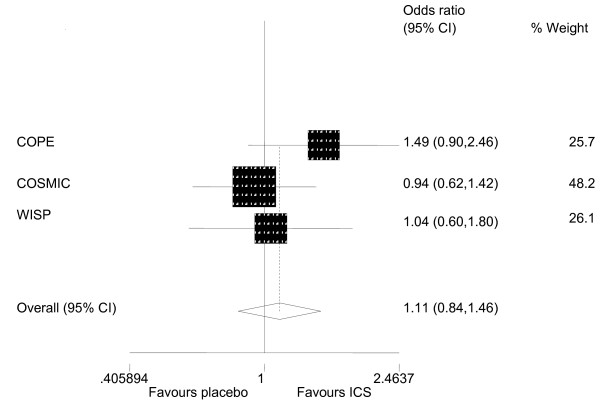
**Meta-analysis of the odds ratio of patients experiencing at least one exacerbation**.

Meta-analysis indicated that patients who had been withdrawn from ICS were slightly more likely to experience an exacerbation in the study period (odds ratio 1.11, 95% CI 0.84 to 1.46), but the effect was not statistically significant (Figure 2). Statistical heterogeneity was not present (P-value = 0.369), but we felt that clinical heterogeneity was. We outline this clinical heterogeneity, and reasons that we had to be cautious about conducting this meta-analysis, in the discussion. Results relating to exacerbations were reported in more than one way in all three parallel trials. In these trials, patients who were withdrawn had more exacerbations than those who were not withdrawn. The difference was significant in COPE [16] but not in WISP [15] or COSMIC [17] (Table 4). In WISP, patients who were receiving placebo experienced an exacerbation on average 19 days earlier than those receiving inhaled steroid (median = 44 days (CI 29-59) *v*. 63 (CI 53-74) days, P = 0.05) [15]. The mean difference in the COPE trial was 34.6 days (95% CI 15.4-53.8) [16]. Change in lung function from baseline was worse in the placebo group in all four trials but only in COSMIC was the difference statistically significant (Table 4).

**Table 4:** Effect size (withdrawn compared with active treatment) 95% confidence intervals and denominators for study outcomes

	*Outcome*													
	
*Study*	Whether exacerbation or not during study period (odds ratio)		Time to first exacerbation (mean (M) or median (m))		Frequency of exacerbation requiring course of steroids or antibiotics(rate ratio (rr) or hazard ratio (hr))		Lung function(mean difference in change from baseline)		Total SGRQ score (mean difference in change from baseline)		Walking distance in 6 minutes (mean difference in change from baseline)		Borg exercise tolerance test (units)	
COPE	1.5	(0.9 to 2.5) (*n *= 242)^a^	34.6 (M)	(15.4 to 53.8) (not clear)	1.5 (hr)	(1.1 to 2.1) (not clear)	-38 ml	(-79.5 to 1.6) (*n *= 242)	2.5	(0.4 to 4.6) (not clear)	9.4 m	(-4.5 to 23.2) (*n *= 172)	-0.3	(-0.7 to 0.3) (*n *= 173)
COSMIC	0.9	(0.6 to 1.4) (*n *= 373)^b^	Not measured		1.2 (rr)	(0.9 to 1.5) (*n *= 373)	-4.1%	(-6.6 to -1.6) (not clear)	0.9	(-.1.3 to 3.1) (not clear)	Not measured		Not measured	
WISP	1.0	(0.6 to 1.8) (*n *= 260)^b^	19 (m)	Not given^c^	1.3 (rr)	(1.0 to 1.6) (*n *= 260)	-23 ml	(-101.5 to 55.5) (not clear)	-0.45	Unable to estimate	Not measured		Not measured	
O'Brien	Not measured		Not measured		Not measured		-11.3%	(-23.4 to 0.8)	Not measured		-32 ft	(-771 to 707)	Unable to estimate^d^	

a all of these exacerbations were moderate - in this trial investigators did not measure exacerbations not requiring treatment

b these exacerbations were mild, moderate or severe

c median = 44 days in withdrawn group (CI 29-59) *v*. 63 (CI 53-74) days in steroid group, P = 0.05

d incorrect confidence interval given in publication.

All three parallel trials [15-17] assessed health related quality of life by means of the St George's Respiratory Questionnaire (SGRQ) [19]. In COSMIC and WISP [15,17] there was no significant difference in the total SGRQ scores, whereas in COPE [16], patients in the placebo group experienced significantly poorer health status in terms of overall score (Table 4). Only COPE and O'Brien measured reported exercise tolerance and there was no evidence that this was affected by withdrawing treatment. In COPE, the use of health care facilities was greater in the placebo group.

## Discussion

There is no conclusive evidence from the trials included in our review that withdrawal of ICS has an effect on the frequency or number of exacerbations. Two trials showed a statistically significant decrease in the time to first exacerbation. Effects on other outcomes are inconclusive. These results are not inconsistent with a recent review in which the authors suggest that the benefits of ICS in preventing COPD exacerbations may be overstated [20].

### Strengths and limitations

We conducted a robust systematic review. Nevertheless, we did not assess publications in languages other than English and, given the small number of studies identified, could not assess publication bias. Limited data within the trial reports meant that we were only able to conduct one meta-analysis.

### Meta-analysis

There are reasons for being cautious about the one meta-analysis we conducted and, in general, meta-analysing results from these types of trial. First, there are differences in patient characteristics between trials, in this case duration of use of ICS and exacerbation rate prior to entry to the trial which may be related to a patient's dependency on inhaled steroids, and differences in outcome definition, in this case definition of exacerbations (Table 5). The two trials conducted most recently also showed less effect of withdrawal which may reflect the increased efficacy of more recent medications which might have been taken alongside the inhaled steroids, although the use of other medication is not reported in detail in the trial reports. An additional reason for being cautious about meta-analysis in trials where medication is withdrawn relates to patient management during the trial, specifically in these trials, the management of exacerbations severe enough to make it unethical for patients to continue on the randomised treatment (Table 4). The WISP study had the highest number of patients discontinuing randomised treatment; 46% in the placebo group and 26% in the fluticasone group. In this trial, a patient's own doctor managed any exacerbation according to usual guidelines. The decision to stop the study inhaler and return to the usual (pre-randomisation) inhaler was made by the patient and doctor without a clearly defined protocol. The procedure was similar in the COSMIC study. By contrast, in the COPE study patients only returned to their usual medication following an examination and decision by one of the trial physicians. Thus, patients in WISP and COSMIC may have had a lower threshold for accepting changes in their normal level of symptoms than in the COPE study and may have left the randomised treatment earlier, especially in the placebo group, resulting in a smaller observed difference in outcome between this group and the fluticasone group in these trials. It is not possible to be prescriptive about a protocol for managing severe exacerbations; different approaches reflect the point of the trial on the pragmatic/explanatory spectrum, and trials at all points are useful in this context. For example, COPE suggests that withdrawing ICS results in an increase in the likelihood of an exacerbation but from WISP there is no indication that doing so in a routine clinical setting with patients participating in the decision to discontinue randomised treatment is likely to result in a change in proportions exacerbating.

**Table 5:** Summary of the way in which trials define and manage exacerbations

	*WISP*	*O'Brien et al*	*COPE*	*COSMIC*
**Definition of exacerbation**	'The presence of at least 2 consecutive days of increase in any two 'major' symptoms (increasing breathlessness, sputum purulence and sputum production" or increase in one 'major' and one 'minor' symptom (wheeze, cough, cold/nasal congestion, sore throat, fever)''Exacerbations were classified as: Unreported: fulfilled symptom criteria on diary cards for a COPD exacerbation but was not managed with antibiotics or oral steroids Moderate: a COPD exacerbation treated with a course of antibiotics or oral steroids Severe: a COPD exacerbation treated with a course of antibiotics or oral steroids resulting in hospital admission'	None	'worsening of respiratory symptoms that required treatment with a short course of oral corticosteroids or antibiotics as judged by the study physician'	'Mild: if a patient on 2 or more consecutive days used 3 or more extra inhalations of salbutamol per 24 hours above their RRV Moderate: if a course of oral steroids were indicated based on a clinician's judgement Severe: if hospitalisation was required'
**Definition of very severe exacerbations which would warrant treatment with medication other than the randomised treatment**	None	None	Rapid recurrent exacerbations: 'twice an objective increase in respiratory symptoms within a three-month period, defined as more than 20% or 300 ml decrease in FEV_1_, compared with stable lung function at randomisation, or 3 times a subjective increase of respiratory symptoms in a 3-month period as experienced by the patient regardless of the criteria mentioned previously'	None
**Protocol for dealing with exacerbations**	'GP's were advised to manage exacerbations according to usual guidance with antibiotics and/or oral steroids. Decisions about stopping study inhalers were made by the general practitioner and patient'	None	'If patients experienced any worsening of symptoms, they were advised to contact the COPE study personnel by phone. They were then invited to attend the hospital within 12 hours for spirometry and consultation by one of the study physicians who decided to continue the trial or to prescribe 500 mcg FP twice daily unblinded'	'Standardised course of prednisolone 30 mg/day for ten days accompanied by a 10 day course of antibiotics.'

### Reporting of individual trials

We recommend that authors make it clear in these trials what other medication patients were likely to have been taking during the trial period and whether there were differences between intervention and control, particularly in situations where the drug being withdrawn is generally seen as an add-on to other medication, as in the case of inhaled corticosteroids. In the COSMIC trial, all patients were prescribed a long acting bronchodilator as part of the trial. In the COPE trial, data on the use of rescue β2 agonists during the study were collected but not presented in the paper. In WISP it is reported that those in the placebo group used a reliever inhaler more frequently during the trial than those in the treatment group. No information is given in the O'Brien trial regarding other medications. Another factor that needs to be clearly reported is the length of time patients have been on inhaled corticosteroids prior to the start of the trial as this may influence the effect of withdrawal; two trials did not give any details of this.

The differences between the trials in the treatment of those experiencing severe exacerbations highlight the need for clear reporting in this area. Currently there is no agreed classification of exacerbations [12]. Providing a clear classification is useful to compare the severity of exacerbations between the groups in a trial, but there needs to be some flexibility in defining events likely to warrant a withdrawal from randomised treatment in these trials. Nevertheless, it is important for authors to report the method of managing exacerbations so that those reading reports are able to interpret results.

A further issue that we became aware of in reviewing these trials is the description of those who discontinue randomised treatment. In the COSMIC trial these individuals were described as drop-outs, although they appeared to provide outcome measures. We suggest that these individuals would be better described as 'discontinuers' and those who do not provide outcome data described as drop outs from the trial. Using this terminology, there were no drop outs in the COSMIC or WISP studies, and only two (both deaths) in the COPE study. There were 134 discontinuers (51%) in the WISP study (56 ICS, 78 placebo), 80 (21%) in COSMIC (34 ICS, 46 placebo) and 32 (13%) in COPE (6 ICS, 26 placebo). Furthermore, taking a pragmatic approach, which is necessary in these trials on ethical grounds, differences in discontinuation between groups are not of paramount importance in assessing the effect of withdrawal.

### Analysis and interpretation of individual trials

How should the results of these trials be analysed and how should these analyses be interpreted? Intention-to-treat (ITT) is the recommended analysis for pragmatic superiority randomised controlled trials [21]. Per protocol analyses were originally suggested as the preferred way to analyse explanatory trials [22], but are now generally regarded as at best an adjunct to ITT analyses because of the potential for selection bias [23]. Here we exemplify this argument for trials where medication is withdrawn.

In such trials, the ethical imperative to allow patients to discontinue randomised treatment and the fact that more in the placebo group usually do so, means that in a per protocol analysis there are likely to be more severely ill individuals in the group receiving ICSs. Thus a per protocol analysis will often underestimate the effect of the withdrawal of treatment on the target population as a whole, and its usefulness in trials at the explanatory end of the spectrum is diminished because an underestimate of effect might lead to treatment being withdrawn when in fact this withdrawal may be harmful. Intention-to-treat analyses make more sense in these trials, but should be interpreted in the context in which the trial is being undertaken. Given the potential differences between the management of patients in different trials, as illustrated in this paper, this interpretation may be unique to the context, and comparisons between different trial results should be made with caution.

Whether there are more suitable analyses for trials in which treatment is withdrawn is an important question. For trials with binary outcomes, Graham Dunn developed a method of allowing for non-compliance while effectively keeping all individuals in an analysis thus overcoming one of the criticisms of per protocol analyses that the balance achieved by randomisation is broken [24]. To our knowledge, there are no publications extending this method to survival analysis, particularly when outcomes are repeated, or to trials in which medication is withdrawn. However, a more important question than the overall effect of withdrawal of treatment on the population may be the identification of patients for whom withdrawal likely to be non-detrimental either in the short or long term.

A further issue in relation to interpretation is the risk of bias introduced by unblinding of patients who have had an exacerbation severe enough to warrant a discontinuation of randomised treatment. It seems likely that there will be more of these patients in the placebo group and this may introduce performance bias.

### Clinical implications

At the moment there are no national or international guidelines advocating the withdrawal of inhaled corticosteroids in those with COPD. Our review does not indicate that patients withdrawn from inhaled corticosteroids will, in general, have substantially worse outcomes than those not withdrawn. Nevertheless, we cannot advocate the withdrawal of inhaled corticosteroids in patients with COPD until further studies confirm which subsets of COPD patients benefit from this treatment and which subsets are at most risk of developing side effects.

## Conclusion

The withdrawal of ICS in patients with COPD may cause exacerbations sooner with a smaller effect on the number of exacerbations. Effects differ according to the definition of outcome and setting and management of patients within the trial. Clarity is needed in the reporting of these trials, particularly in defining exacerbations, reporting the management of exacerbations severe enough to warrant discontinuation of randomised treatment, the description of individuals who discontinue, and the collection and analysis of their outcomes. ITT analyses are appropriate for these trials, but other methods of analysis could be explored including analyses which attempt to identify those for whom discontinuation results in little in the way of detrimental effects.

## List of Abbreviations

COPD: chronic obstructive pulmonary disease; ICS: inhaled corticosteroids; NICE: National Institute for Clinical Excellence; ATS: American Thoracic Society; GOLD: Global initiative for chronic obstructive pulmonary disease; COPE: Effects of discontinuing inhaled corticosteroids in patients with chronic obstructive pulmonary disease; COSMIC: COPD and Seretide: a multi-centre intervention and characterisation; WISP: Withdrawal of inhaled corticosteroids in people with COPD; SGRO: St George's respiratory questionnaire; FEV: forced expiratory volume.

## Competing interests

The authors declare that they have no competing interests.

## Authors' contributions

SE conceived the study. SE and NN designed the study. NN identified trials. All three authors assessed the quality of the trials. SE and NN extracted data from the trials. SE performed the meta-analysis. All three authors wrote and revised the manuscript.

## References

[B1] Global Initiative for Obstructive Lung Disease Global strategy for the diagnosis, management, and prevention of chronic obstructive pulmonary disease 2006 http://www.goldcopd.com/(accessed 22 April 2007)

[B2] Murray CJ Lopez AD Alternative projections of mortality and disability by cause 1990-2020: Global Burden of Disease Study Lancet 1997 349 1498 1504 10.1016/S0140-6736(96)07492-2 9167458

[B3] National Institute for Clinical Excellence Chronic obstructive pulmonary disease 2004 http://www.nice.org.uk/CG101 (last accessed 29 July 2011)

[B4] British Thoracic Society (BTS) Burden of Lung Disease Report 2006 2 http://www.brit-thoracic.org.uk/delivery-of-respiratory-care/burden-of-lung-disease-reports.aspx (last accessed 29 July 2011)

[B5] Calverley PMA Anderson JA Bartolome C Ferguson GT Jenkins C Jones PW Vestbo J Salmeterol and Fluticasone Propionate and Survival in Chronic Obstructive Pulmonary Disease N Engl J Med 2007 356 775 89 10.1056/NEJMoa063070 17314337

[B6] Yang IA Fong K Sim EHA Black PN Lasserson TJ Inhaled corticosteroids for stable chronic obstructive pulmonary disease Cochrane Database of Systematic Reviews 2007 2 Art. No.: CD002991 10.1002/14651858.CD002991.pub2 17443520

[B7] Celli BR Thomas NE Anderson JA Ferguson GT Jenkins CR Jones PW Vestbo J Knobil K Yates JC Calverley PM Effect of pharmacotherapy on rate of decline of lung function in chronic obstructive pulmonary disease: results from the TORCH study Am J Respir Crit Care Med 2008 178 4 332 8 10.1164/rccm.200712-1869OC 18511702

[B8] Drummond MB Dasenbrook EC Pitz MW Murphy DJ Fan E Inhaled Corticosteroids in Patients With Stable Chronic Obstructive Pulmonary Disease: A Systematic Review and Meta-analysis JAMA 2008 300 2407 2416 10.1001/jama.2008.717 19033591 PMC4804462

[B9] Cumming RG Mitchell P Leeder SR Use of inhaled corticosteroids and the risk of cataracts N Engl J Med 1997 337 8 14 10.1056/NEJM199707033370102 9203425

[B10] Garbe E LeLorier J Boivin J Suissa S Inhaled and nasal glucocorticocoids and the risk of ocular hypertension or open-angle glaucoma JAMA 1997 277 722 727 10.1001/jama.277.9.722 9042844

[B11] Weatherall M James K Clay J Perrin K Masoli M Wijesinghe M Beasley R Dose response relationship for risk of non-vertebral fracture with inhaled corticosteroids Clin Exp Allergy 2008 38 9 1451 145 10.1111/j.1365-2222.2008.03029.x 18537983

[B12] American Thoracic Society Standards for the diagnosis and management of patients with COPD 2004 http://www.thoracic.org/clinical/copd-guidelines/(last accessed 29 July 2011)

[B13] Higgins JPT Green S editors Cochrane Handbook for Systematic Reviews of Interventions 5.0.2 http://www.cochrane.org/training/cochrane-handbook (last accessed 29 July 2011), [updated September 2009]

[B14] DerSimonian R Laird N Meta-analysis in clinical trials Control Clin Trials 1986 7 3 177 88 10.1016/0197-2456(86)90046-2 3802833

[B15] Choudhury AB Dawson CM Kilvington HE Eldridge S James W Wedzicha JA Feder GS Criffiths CJ Withdrawal of inhaled corticosteroids in people with COPD in primary care: a randomised controlled trial *Respiratory Research* 2007 8 93 10.1186/1465-9921-8-93 PMC2245934 18162137

[B16] van der Valk P Monninkhof E van der Palen J Zielhuis G van Herwaarden C Effects of discontinuing inhaled corticosteroids in patients with chronic obstructive pulmonary disease. The COPE study Am J Respir Crit Care Med 2002 166 1358 1363 10.1164/rccm.200206-512OC 12406823

[B17] Wouters EFM Postma DS Fokkenst B Hop WCJ Prins J Kuipers AF Pasma HR Hensing CAJ Creutzberg EC for the COSMIC (COPD and Seretide: a multi-centre intervention and characterisation) study group Thorax 2005 60 480 487 10.1136/thx.2004.034280 15923248 PMC1747438

[B18] O'Brien A Russo-Magno P Karki A Hiranniramol S Hardin M Kaszuba M Sherman C Rounds S Effects of withdrawal of inhaled steroids in men with severe irreversible airflow obstruction Am J Respir Crit Care Med 2001 164 3 365 371 11500334 10.1164/ajrccm.164.3.2002052

[B19] Jones PW Quirk FH Baveystock CM The St George's Respiratory Questionnaire Respir Med 1991 85 Suppl B 25 31 discussion 33-7 1759018 10.1016/s0954-6111(06)80166-6

[B20] Agarwal R Aggarwal AN Gupta D Jindal SK Inhaled corticosteroids vs placebo for preventing COPD exacerbations: a systematic review and metaregression of randomized controlled trials Chest 2010 137 2 318 25 10.1378/chest.09-1305 19783669

[B21] Moher D Schulz KF Altman DG CONSORT Group (Consolidated Standards of Reporting Trials) The CONSORT statement: revised recommendations for improving the quality of reports of parallel-group randomized trials J Am Podiatr Med Assoc 2001 91 8 437 42 11574648 10.7547/87507315-91-8-437

[B22] Schwartz D Lellouch J Explanatory and pragmatic attitudes in therapeutical trials Journal of Chronic Diseases 1967 20 637 648 10.1016/0021-9681(67)90041-0 4860352

[B23] Peto R Collins R Gray R Large-scale randomized evidence: large, simple trials and overviews of trials Journal of Clinical Epidemiology 1995 48 1 23 40 10.1016/0895-4356(94)00150-O 7853045

[B24] Dunn G Maracy M Dowrick C Ayuso-Mateos JL Dalgard OS Page H Lehtinen V Casey P Vazquez-Barquero JL Wilkinson G ODIN group Wilkinson C Estimating psychological treatment effects from a randomised controlled trial with both non-compliance and loss to follow-up Br J Psychiatry 2003 183 323 31 10.1192/bjp.183.4.323 14519610

